# Supplementary material for: Findings on emergent magnetic resonance imaging in pregnant patients with suspected appendicitis: A single center perspective
Source: PLoS One. 2024 Feb 8;19(2):e0288156. doi: 10.1371/journal.pone.0288156 (PMC10852244; doi:10.1371/journal.pone.0288156)
Supplement: Attachment — Submitted filename: response to reviewers.docx [file pone.0288156.s001.docx]

Dear Doctor Reisman,

Please find below a point by point response to the reviewers' comments.

The reviewers’ comments greatly improved our study and we hope that it is now fit for publication in you esteemed journal.

**Reviewer #1:**

Major comments
**The scan parameters in the MR protocol are insufficient (i.e., lack of data except for TR and TE). The parameters must be more detailed to allow other investigators to reproduce them.**

As suggested, we've added the following parameters to the MRI protocol table (Table 1): slice thickness, gap and matrix. **The formal reading session with an independent reading by subspecialized GI or ER radiologists is preferable for this study design. The design of this study does not show inter-reader disagreement and differences between radiologists with different experience years.**

As suggested, we've added to the results a kappa value in order to clarify inter-reader agreement: " There were five cases of disagreement between readers for which the final categorization was assigned according to the senior radiologist’s reading. Cohen’s Kappa for inter-reader agreement was 0.9 (CI 0.818-0.916)." **It is unknown how the final diagnosis was determined. The authors would clarify how many specific diagnoses were made and which evidence (surgery, pathology, clinical course, etc…)**

Thank you for your comment. We have added a clarification to the Methods section regarding final diagnosis: "Final diagnosis was determined according to pathological reports if patient underwent surgery, or clinical improvement if patient was managed only medically."

**Minor comments
“gastrointestinal” is better than “surgical” (P4L5)**

This was changed according to your suggestion.

**Oral contrast administration is risky for GI symptoms even if the patients were clinically suspected of acute appendicitis. Some patients with bowel obstruction complain of right lower abdominal pain. (P4L14)**

Thank you for this important comment.

As mannitol is a class C contrast agent during pregnancy [1], there is no evidence of any adverse fetal effects. As to possible side effects, in a study evaluating side effects caused by mannitol digestion, the severity of side effects was related to the amount of mannitol administered. With an amount of 1000 ml (similar to that used in our study), gastrointestinal side effects were uncommon [2].

[1] Product Information: ARIDOL(TM) inhalation powder, mannitol inhalation powder. Pharmaxis, Inc, Exton, PA, 2010.
[2] Ajaj, W., Goehde, S.C., Schneemann, H., Ruehm, S.G., Debatin, J.F. and Lauenstein, T.C. (2004), Dose optimization of mannitol solution for small bowel distension in MRI. J. Magn. Reson. Imaging, 20: 648-653.

**Please describe more details for the statistical approach.**

The statistical approach was described in further detail in the Methods section:. " Continuous parameters are presented as median (interquartile range (IQR)). Statistical analysis was performed using SPSS Statistics software (version 27.0, IBM), proportions were described as percentage. Mean and median were calculated. Kappa rate was calculated according to Cohen's Kappa, and is presented with confidence interval (CI)."


**There is no definition for definite and indeterminate acute appendicitis in US.**

Definite appendicitis was diagnosed as accepted in radiological literature [3,4] as appendicular width > 6 mm, lack of compressibility, inflamed echogenic periappendiceal fat. Indeterminate appendicitis was defined as one or more of the following: inflamed echogenic periappendiceal fat, right lower quadrant fluid collection or enlarged mesenterial lymph nodes without the visualization of a widened and non-compressible appendix. This was also added to the Methods section.

# [3] Yu SH, Kim CB, Park JW, Kim MS, Radosevich DM.   Ultrasonography in the Diagnosis of Appendicitis: Evaluation by Meta-analysis.   Korean J Radiol. 2005 Oct-Dec;6(4):267-277. [4] Barbara Hertzberg, William Middleton. Ultrasound: The Requisites, 3rd Edition - July 13, 2015.

**Results
No 95% IC for sensitivity and specificity.**
As the examples of sensitivity and specificity were derived from the literature which did not contain CIs, we could not provide this data.

 **The patient demographics are recommended to be summarized in a Table.**

As suggested, table 2 was added to the manuscript and contains patient demographics.

**Discussion
The authors could discuss the results in other aspects (e.g., the discrepancy between the results on US and MR in each category [gastrointestinal, gynecological, and urological disease]).**

Thank you for this comment. We added to the Discussion section the following paragraph: “Although our study did not focus on US in the settings of acute RLQ pain in the pregnant patient, it is noticeable that alternative diagnoses proposed on MRI were not diagnosed on US, such as ruptured ectopic pregnancy or hematosaplinx, pyelonephritis or incarcerated umbilical hernia. As US in the pregnant patient has limitations due to patient habitus and physiological changes, and as MRI is readily available in our intuition for these patients, final patient management was determined according to a combination of clinical and MRI findings in all cases. This can emphasize the usefulness of MRI in pregnant patients with RLQ pain, compared to using US only. “

Table
**It is unclear the difference between “no pathological findings” and “normal appendix identified” in Table 2.**

We changed the term "no pathological findings" to "Appendix was not visualized and there were no other findings to account for abdominal pain.”

**Reviewer #2:**

**There is no table with description of the population included in the study and the clinical investigations and characteristics. A figure with a flow diagram would be useful.**

Please find our response to reviewer 1, comment 9 (new Table 2). We have also added a flow chart as figure 1.

**Were initial gynecological ultrasound performed? It is rather surprising an ectopic pregnancy was diagnosed by MR.**

Yes, all patients underwent gynecological US prior to MRI. We added a clarification in the Methods section: "All patients underwent gynecological US prior to MRI and were only referred to further imaging if gynecological US did not find a cause of abdominal pain. "

**The manus would benefit from a rewriting with clinical perspective and MR findings.**

As suggested, the paper was reviewed and some clinical aspects were added.
