# Supplementary material for: Findings on emergent magnetic resonance imaging in pregnant patients with suspected appendicitis: A single center perspective
Source: PLoS One. 2024 Feb 8;19(2):e0288156. doi: 10.1371/journal.pone.0288156 (PMC10852244; doi:10.1371/journal.pone.0288156)
Supplement: Attachment — Submitted filename: Response to Reviewers.docx [file pone.0288156.s002.docx]

Dear Dr. Reisman,

Thank you for reviewing our paper.
Please find below a point by point response to the reviewer's comments.

The reviewer's comments greatly improved our study and we hope that it is now fit for publication in you esteemed journal.

**Reviewer #3:
In most institutions there is always a shortage of MRI capacity. The authors present a rather complex MR program. How long is the examination time? Are there essential sequences allowing a faster examination? Is there a 10 Minute examination possible?**

Our medical center is a large tertiary center, the largest in the Middle East, and we have the privilege of having multiple MRI machines available, and therefore we are always able to accommodate emergent MRI scans whenever required, around the clock. As to a possible abbreviated MRI protocol, our study did not focus on optimizing the complex protocol and are unable to assess whether a shorter protocol is feasible. In our clinical experience of over a decade of emergent MR imaging for possible acute appendicitis in pregnant patients, there is no single sequence which can serve as an optimal sequence to visualize the appendix, and in every patient the appendix is best visualized in different sequences. For that reason, and the readily available MRI time, we do decided not use an abbreviated MRI protocol, and our protocol takes 28 minutes to perform. Further future studies are required to validate such abbreviated MRI protocol.

**The authors should comment on the safety of MRI for pregnant patient and the baby for the non radiologist reader. And they should comment on the contraindication of gadolinium based contrast media especially in the first trimester.**

Thank for this important comment. We have added the following explanation in the *introduction* section: " According to the American College of Gynecology (ACOG), Non-contrast MRI is safe to use in pregnant patients in all trimesters, including first trimester. Gadolinium is water soluble and may cross the placenta to the amniotic fluid and was found to be teratogenic in animal studies thus it is contraindicated in the pregnant patients."

**A crucial question is the outcome of the patients without a diagnosis on MRI. How was the patient outcome assessed? Has there been a systematic chart review? It would be interesting how many of the patients with negative MRI showed cystitis, a diagnosis usually not detectable by MRI. A more systematic report of the patients outcome would greatly enhance the paper.**

Most patients who did not have a clear diagnosis on MRI were admitted to the high risk pregnancy unit and were discharged after a clinical improvement, some without any specific treatment and some with the use of empiric antibiotics. Given the nature of our local health system, electronic patient files for community follow-up are not available to us, and in most cases we indeed did not have a long period follow up. The few patients who were followed in our hospital clinics had a long follow up available to us. Overall, only one patient was discharged with the diagnosis of cystitis, not seen on MRI. We have added to the results section the data related to UTI and the following paragraph regarding long term follow up: "As most obstetric patients in our healthcare system are not followed in hospital but rather in community-based clinics not affiliated to our hospital, we do not have access to long term follow up for most patients. However, as delivery data is available to us, we have found that none of the patients who presented to hospital with RLQ pain and did not have acute appendicitis had a preterm delivery or any other obstetric complications, regardless of whether the MRI found a cause for their pain or not."
